# Synergistic antitumour activity of HDAC inhibitor SAHA and EGFR inhibitor gefitinib in head and neck cancer: a key role for ΔNp63α

**DOI:** 10.1038/s41416-019-0394-9

**Published:** 2019-02-15

**Authors:** Simona Citro, Alice Bellini, Claudia Miccolo, Lavinia Ghiani, Thomas E. Carey, Susanna Chiocca

**Affiliations:** 1 Department of Experimental Oncology, IEO, European Institute of Oncology IRCCS, Via Adamello 16, 20139 Milan, Italy; 2 Department of Otolaryngology-Head and Neck Surgery, Department of Pharmacology, Comprehensive Cancer Center, University of Michigan Medical School, Ann Arbor, MI USA

**Keywords:** Head and neck cancer, Cancer

## Abstract

**Background:**

Epidermal growth factor receptor (EGFR) overexpression is associated with the development of head and neck cancer (HNC) and represents one of the main therapeutic targets for this disease. The use of EGFR inhibitors has limited efficacy due to their primary and acquired resistance, partially because of increased epithelial to mesenchymal transition (EMT). The HDAC inhibitor SAHA has been shown to revert EMT in different tumours, including HNC. In this study, we investigated the cooperative role of SAHA and the EGFR tyrosine kinase inhibitor gefitinib in both HPV-positive and HPV-negative HNC cell lines.

**Methods:**

A panel of 12 HPV-positive and HPV-negative HNC cell lines were screened for cell viability upon treatment with SAHA, gefitinib and the combination of the two. Epithelial/mesenchymal marker expression, as well as activation of signalling pathway, were assessed upon SAHA treatment. ΔNp63α silencing with shRNA lentiviral particles was used to determine its role in cell proliferation, migration and TGFβ pathway activation.

**Results:**

We found that both SAHA and gefitinib have antitumour activity in both HPV-positive and HPV-negative HNC cell lines and that their combination has a synergistic effect in inhibiting cell growth. SAHA treatment reverts EMT and inhibits the expression of the transcription factor ΔNp63α. Suppression of ΔNp63α reduces EGFR protein levels and decreases cell proliferation and TGFβ-dependent migration in both HPV-positive and HPV-negative HNC cell lines.

**Conclusions:**

Our results, by giving a clear molecular mechanism at the basis of the antitumour activity of SAHA in HNC cell lines, provide a rationale for the clinical evaluation of SAHA in combination with gefitinib in both HPV-positive and HPV-negative HNC patients. Further knowledge is key to devising additional lines of combinatorial treatment strategies for this disease.

## Background

Head and neck cancer (HNC) includes malignant squamous lesions arising in the oral cavity, oropharynx, larynx or hypopharynx, and is the sixth leading cancer by incidence worldwide, with approximately 500,000 new cases annually and only 40–50% 5-year survival rate.^1^ The use of tobacco and excessive alcohol consumption are the most important risk factors identified and they also seem to have a synergistic effect. A subgroup of HNC, particularly those of the oropharynx, is caused by infection with high-risk types of human papillomavirus (HPV). Although in the past decade the incidence of HNC has been slowly declining in the western world, probably due to a decrease in the prevalence of smoking, oral tongue and particularly oropharyngeal cancers are becoming more prevalent. This may be related to an increase in oropharyngeal HPV infections and recent studies revealed that HPV16 in particular is involved in these tumours. HPV-positive tumours form a distinct group within HNC with different aetiological factors. These tumours are different at the molecular level, changing the clinical outcome and presenting a better prognosis.^2^ Despite their diversities, HPV-positive and HPV-negative HNC patients are treated using the same therapies, leading the scientific and medical community to reassess the current treatment protocols, in order to develop less toxic strategies while maintaining good oncological outcomes. Recently, the anti-PD-1 antibodies nivolumab and pembrolizumab have been approved as targeted therapies for the treatment of HNC,^3^ as well as cetuximab. Cetuximab, an anti-epidermal growth factor receptor (EGFR) monoclonal antibody, has been approved for the treatment of HNC in patients with locally advanced tumours in association with radiotherapy and in patients with recurrent or metastatic diseases in combination with cisplatin-based chemotherapy.^4,5^ Small-molecule tyrosine kinase inhibitors (TKIs) such as gefitinib, which prevent the binding of ATP to the receptor and thereby inactivate EGFR, have also been investigated for HNC in preclinical and clinical settings, showing tumour growth delay and enhancement of apoptosis^4^ and single-agent activity in recurrent and metastatic disease.^6^ Although overexpression of EGFR has been observed in about 90% of HNC specimens and correlates with poor prognosis, advanced disease and reduced survival, cetuximab treatment has yielded only modest clinical outcomes as a monotherapy.^7^ Taken together, this suggests that primary and acquired resistance mechanisms considerably limit the clinical benefit of cetuximab in HNC,^8^ which translates in a clear need to understand the molecular mechanisms driving cetuximab resistance to maximise the treatment response by patient selection and to establish new treatment options to overcome resistance.

Epithelial to mesenchymal transition (EMT) is known to be deeply involved in cancer progression and metastasis. EMT is characterised by the loss of proteins involved in cell junctions such as E-cadherin, and the expression of mesenchymal markers such as vimentin.^9^ Acquisition of EMT features has also been associated with chemoresistance acquired after standard chemotherapy.^10^ Activation of both transforming growth factor β (TGFβ) signalling and receptor tyrosine kinase plays critical roles in EMT either through the downstream SMAD signalling or through a SMAD-independent pathway, such as activation of ERK MAP kinases, Rho GTPases and the PI3 kinase/Akt pathway.^11^ Cancer cells can increase their production of active TGFβ during development of EGFR-TKI resistance.^12^ TGFβ triggers EMT and allows the cells to become invasive. EMT seems to also play a role in establishing the resistance to EGFR TKIs in HNC.^13^

In the last years, histone deacetylase inhibitors (HDACi) have been described as valid tools to overcome EMT in many different tumours,^12,14,15^ including HNC,^16^ whereas in other types of cancer the use of HDACi has been shown to increase EMT.^17–19^ HDACs are typically overexpressed in cancers, such as HNC,^20^ altering gene transcription and enhancing cell proliferation. To date, several HDACi have been developed and used in clinical trials for the treatment of cancers, and have been shown to induce differentiation, growth arrest or apoptosis in tumour cells.^21,22^ Currently, the US Food and Drug Administration has approved four types of HDACi for cancer therapy, including vorinostat (SAHA, Zolinza)^23^ for the treatment of refractory cutaneous T-cell lymphoma (CTCL), romidepsin (Istodax)^24^ for the treatment of CTCL and peripheral-cell lymphoma (PTCL), belinostat (Beleodaq) for the treatment of PTCL^25^ and panobinostat (Farydak) for the treatment of multiple myeloma.^26,27^ Recently, HDACi have been shown to reduce ΔNp63α protein stability in two cutaneous squamous cell lines.^28^ The p63 isoform ΔNp63α, member of the p53 family, is a transcription factor essential for terminal differentiation of stratified epithelia.^29^ ΔNp63α is the predominant p63 isoform expressed in normal squamous epithelia and squamous carcinomas and its expression is upregulated in up to 80% of primary HNC tumours.^30^ Many studies have highlighted the oncogenic potential of ΔNp63α in HNC promoting squamous epithelial proliferation, migration and inflammation,^31,32^ and regulating EMT in primary human keratinocytes and in HNC cell lines in a TGFβ-dependent manner.^33,34^ Moreover, HDACi, alone or in combination, have been shown to reduce EGFR expression in HNC cell lines.^16,35^

In this study we investigated the molecular mechanisms at the basis of the antitumour activity of the HDACi SAHA in HNC cell lines. We demonstrated that SAHA possesses a synergistic inhibitory effect in combination with gefitinib, which is neither dependent on the HPV status nor on the epithelial/mesenchymal phenotype of the cell lines. We then demonstrated that the mechanism by which SAHA controls cell proliferation and reverts EMT is due to its ability to decrease the expression of the transcription factor ΔNp63α. Thus, with our data we uncover new molecular mechanisms underlying the potential efficacy in the clinic of the use of SAHA in combination with gefitinib. Indeed, the use of the two inhibitors together have greater effect in blocking cancer cell proliferation compared to single treatment and might avoid gefitinib resistance in HNC cancer cells, due to the ability of SAHA to revert EMT.

## Materials and methods

### Cell culture, reagents and plasmids

HNC cell lines were obtained from different sources.^36^ The UM-SCC-4, UM-SCC-6, UM-SCC-10A, UM-SCC-18, UM-SCC-19, UM-SCC-23 and UM-SCC-47 cell lines were created by Prof. Thomas E. Carey.^36,37^ The UD-SCC-2 cell line^37^ was kindly provided by Prof. Henning Bier: present address LRZ, Munich, Germany. The 93-VU-147T cell line^38^ was kindly provided by Dr. Martin Rooimans, Free University Medical Centre, Amsterdam, the Netherland. The UM-SCC-104 cell line^39^ was from Merck spa. The UPCI:SCC-154^40^ and UPCI:SCC-90^40,41^ cell lines were acquired from ATCC^®^. Cells were grown in Dulbecco’s modified Eagle’s medium supplemented with antibiotics, 2 mM l-glutamine, 10% foetal bovine serum and non-essential amino acids. All cell lines were authenticated by short tandem repeat profiling and tested for mycoplasma contamination every 6 months. Recombinant human TGFβ1 was purchased from PeproTech, SAHA from Alexis Biochemicals and gefitinib (ZD1839, Iressa^®^) from Sigma Aldrich. Lentiviral plasmid encoding shControl and shp63 were a kind gift from Dr. Leif W.Ellisen (Massachusetts General Hospital Cancer Center and Harvard Medical School, Boston, Massachusetts). pBABE retroviral plasmid encoding EGFR was a kind gift of Dr. Sara Sigismund (European Institute of Oncology, Milan, Italy).

### Cells lysis and western blot analysis

Cells were lysed in either a sodium dodecyl sulphate (SDS) lysis buffer: a 1:3 mixture of buffer I (5% SDS, 0.15 M Tris-HCl [pH 6.8] and 30% glycerol) and buffer II (25 mM Tris-HCl [pH 8.3], 50 mM NaCl, 0.5% NP-40, 0.1% SDS, 1 mM EDTA and protease inhibitors) containing 0.5 mM *N*-ethylmaleimide (NEM), 0.5 mM NaF and 2 mM sodium Na_3_VO_4_, or in E1A buffer (50 mM HEPES [pH 7], 250 mM NaCl, 0.1% NP-40, proteases inhibitors, 0.5 mM NEM, 0.5 mM NaF and 2 mM Na_3_VO_4_). After lysis an equal amount of protein for each sample was resuspended in denaturing sample loading buffer, separated on SDS-polyacrylamide gel electrophoresis gel and immunoblotted with the indicated antibodies. The following antibodies were used: E-Cadherin (rabbit, Cell Signaling), Vimentin (mouse, Abcam), p63 (mouse, Abcam), acetyl-histone3 (Lys9) (rabbit, Upstate), acetyl-histone4 (Lys8) (rabbit, Abcam), phospho-SMAD2 (rabbit, Cell Signaling), EGFR (rabbit, homemade), phospho-EGFR (tyr1068) (rabbit, Cell Signaling), acetylated-αtubulin (mouse, Sigma), phospho-p70 S6 kinase (rabbit, Cell Signaling), phospho-Akt (rabbit, Cell Signaling), phospho-p38 (rabbit, Cell Signaling), phospho-Erk (rabbit, Cell Signaling), HPV16 E7 (mouse, Santa Cruz) and GAPDH (mouse, Abcam), and Vinculin (mouse, Sigma Aldrich) as loading control. Membranes were then incubated with the appropriate horseradish peroxidase secondary antibodies and the signal was acquired with Chemidoc (Bio-Rad).

### Quantitative reverse transcription PCR (RT-qPCR)

RNA was extracted from cells with the Quick-RNA MiniPrep kit (Zymo Research). cDNA was generated by reverse transcription-PCR with Reverse Transcriptase (Promega). Relative levels of specific mRNAs were determined with the Fast SYBR Green detection chemistry system (Applied Biosystem). All PCR reactions were performed with a 7500 Fast Real-Time PCR system (Applied Biosystem). Ribosomal phosphoprotein was used as a house-keeper gene for normalisation.

### Cell proliferation assay

p63 short hairpin RNA-encoding lentiviral particles were used to transduce HNC cell lines. After puromycin selection, cells were plated in triplicate into 96-well plates at the appropriate density (UD-SCC-2 at 2000 cells/well; UM-SCC-4, UM-SCC-6, UM-SCC-10A, UM-SCC-104 and UPCI:SCC-90 at 1666 cells/well). Cell proliferation was assayed at day 3 and 7 after plating (T0) (UM-SCC-4, UM-SCC-6, UM-SCC-10A and UPCI:SCC-90) or at day 2 and 6 (UM-SCC-104 and UD-SCC-2), using CellTiter-Glo^®^ Luminescent Cell Viability Assay and following the manufacturer’s instructions.

### Half maximal inhibitory concentration analysis

To assess the half maximal inhibitory concentration (IC50) of SAHA and gefitinib, HNC cell lines were seeded in duplicate at the appropriate density (UD-SCC-2 at 6000 cells/well; UM-SCC-18 and UM-SCC-19 at 4000 cells/well; UM-SCC-4, UM-SCC-6, UM-SCC-10A, UM-SCC-23, UM-SCC-47, UM-SCC-104, UPCI:SCC-90, UPCI:SCC-154 and 93-VU-147T at 5000 cells/well) in 96-well plates. Twenty-four hours later cell lines were treated with vehicle or different concentrations of SAHA (1:3) (0.21, 0.62, 1.85, 5.56, 16.67 and 50 μM) and (1:2.5) gefitinib (0.06, 0.16, 0.41, 1.02, 2.56, 6.40, 16.00, 40.00 and 100.00 μM) using serial dilutions, for 72 h. Cell proliferation was assayed using CellTiter-Glo^®^ Luminescent Cell Viability Assay and following the manufacturer’s instructions. Data were analysed using Graphpad Prism software.

### Drug combination studies

HNC cell lines were seeded at the appropriate density (UD-SCC-2 at 6000 cells/well; UM-SCC-4, UM-SCC-23 and UPCI:SCC-154 at 5000 cells/well) in duplicate in 96-well plates. At 24 h, cell lines were treated with vehicle or different concentrations of SAHA, gefitinib or the combination of the two drugs in equi-active concentrations using serial dilutions in a 1:1 constant ratio (0.16, 0.41, 1.02, 2,56, 16.00 and 40 μM) for 72 h. Cell proliferation was assayed with CellTiter-Glo^®^ Luminescent Cell Viability Assay, following the manufacturer’s instructions. The combination index (CI) was calculated by the Chou-Talalay equation, which takes into account both potency (Dm or IC50) and the shape of the dose-effect curve^42^ using the software CalcuSyn (Biosoft, Cambridge, UK). CI < 1, CI = 1 and CI > 1 indicate synergism, additive effect and antagonism, respectively. The linear correlation coefficient (*r*) of the median-effect plot is considered the first line of statistics to measure the conformity of the data with the mass-action law principle when the experimental measurement is assumed to be accurate. An *r*-value = 1 indicates perfect conformity. A poor *r*-value may be the result of biological variability or experimental deviations. Dose reduction index represents the measure of how much the dose of each drug in a synergistic combination may be reduced at a given effect level compared with the doses of each drug alone.

### Migration assay

Migration was evaluated by wound-healing assay. Briefly, HNC cells were seeded to 90% of confluence in 35 mm cell culture dishes with grid. Twenty-four hours after plating, the cell monolayer was wounded in two different points with a sterile pipette tip. After washing out the floating cells, cells were rinsed with fresh medium. Migration of wounded cells was observed and photographed at 0 and 24 h with an optic microscope. Photographs were taken at 0 and 24 h after wounding by brightfield and phase contrast microscopy (Evos fl, Advanced Microscopy Group, Inc.). Quantitative measurements were made by determining the distances between the wound edges in at least three independent wound sites. The migration values were obtained by using ImageJ software (National Institute of Health, USA), and expressed as % of migration respect to time points 0 h of culture.

### Statistical analysis

Statistical differences were evaluated using Dunnet’s multicomparison analysis after one-way analysis of variance to compare multiple samples or unpaired *t* test to compare only two samples (Graphpad Prism version 6 software).

## Results

### Antiproliferative effect of SAHA and gefitinib and their synergistic activity in both HPV-positive and HPV-negative HNC cell lines

We screened the effect of both SAHA and gefitinib on cell viability in a panel of 12 HNC cell lines, 6 of them deriving from HPV-positive patients (Table S1).^43^ As shown in Table 1, cells were differentially sensitive to SAHA and gefitinib independently of the HPV status. In particular, the UPCI:SCC-90 and UD-SCC-2 cell lines responded differently upon drug treatment, despite they are both HPV-positive and have a mesenchymal phenotype as shown by the E-cadherin and vimentin expression levels (Figure S1A). Moreover, treating the cell lines most resistant to gefitinib, upon combination of SAHA and gefitinib, we could clearly appreciate a synergistic effect of the two drugs together, independently from the HPV status (Table 2, CI index). Thus, we showed that SAHA and gefitinib have an inhibitory and synergistic activity in HNC cell lines, which seems neither related to the HPV status of HNC cell lines nor to their epithelial/mesenchymal phenotype.

**Table 1:** Half maximal inhibitory concentration values for SAHA and gefitinib (μM)

Cell lines	IC50 SAHA	IC50 gefitinib	HPV status
UM-SCC-4	2.23	2.51	−
UM-SCC-6	3.46	0.04	−
UM-SCC-10A	4.74	0.47	−
UM-SCC-18	0.84	0.0089	−
UM-SCC-19	2.44	0.98	−
UM-SCC-23	1.04	2.57	−
UM-SCC-47	3.75	8.43	+
UPCI:SCC-90	1.55	0.37	+
UM-SCC-104	1.84	0.058	+
UPCI:SCC-154	2.44	14.3	+
UD-SCC-2	5.28	6.96	+
93-VU147T	2.32	0.13	+

Mean of at least three different experiments done in duplicate

*IC50* half maximal inhibitory concentration, *HPV* human papillomavirus

**Table 2:** Combination index and dose reduction index values for SAHA and gefitinib combination (μM)

Cell lines	CI50	DRI50	*r*
UM-SCC-4	0.40981	Gefitinib: 4.92263 SAHA: 4.83860	0.94028
UM-SCC-23	0.21784	Gefitinib: 14.0835 SAHA: 6.81026	0.96417
UPCI:SCC-154	0.26621	Gefitinib: 29.9400 SAHA: 4.29528	0.92685
UD-SCC-2	0.11638	Gefitinib: 13.5178 SAHA: 23.5849	0.96664

Mean of at least three different experiments done in duplicate. CI and DRI values computed at 50% of cell death. CI < 1, CI = 1 or CI > 1 generally indicate synergistic, additive or antagonistic effect. DRI values represent the order of magnitude (fold) of dose reduction obtained for IC50 (DRI50) in combination setting compared with each drug alone. *r* is the coefficient of correlation for the fitting between CIs and fractional effects.

*CI* combination index, *DRI* dose reduction index

### SAHA treatment reverts EMT in both HPV-positive and HPV-negative HNC cell lines, inhibits TGFβ pathway activation and decreases the expression of ΔNp63α

**Fig. 1:**
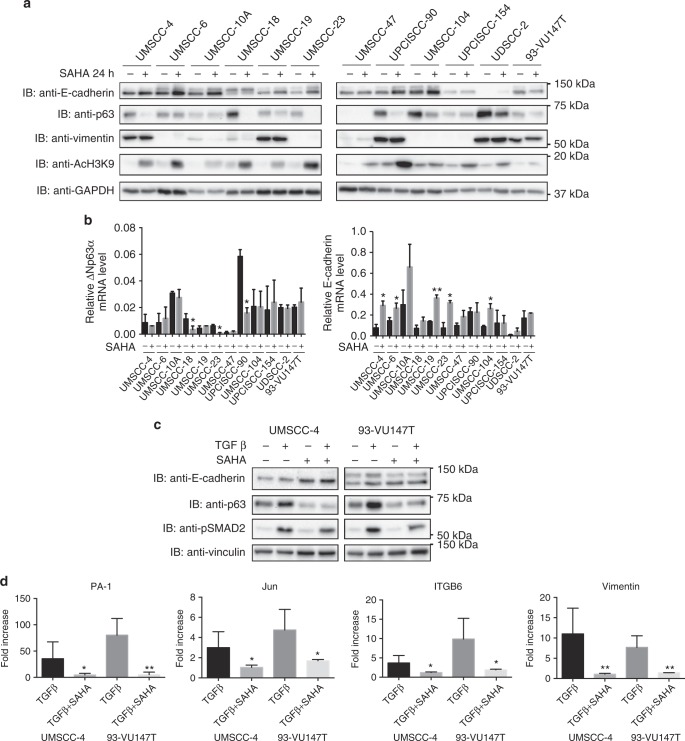
SAHA treatment reverts epithelial to mesenchymal transition in human papillomavirus (HPV)-negative and HPV-positive head and neck cancer (HNC) cell lines and inhibits transforming growth factor β (TGFβ) activity. **a** HNC cell lines were treated with 5 μM SAHA or vehicle for 24 h, lysed and analysed by immunoblotting (IB) with the indicated antibodies. **b** Total RNAs from HNC cell lines treated with 5 μM SAHA or vehicle for 24 h were isolated for RT-qPCR. ΔNp63α, E-cadherin expression was normalised to ribosomal phosphoprotein (RpPO) and expressed as means ± SD of at least three independent experiments. *P < 0.05; **P < 0.01 (unpaired *t* test). **c** HNC cell lines were serum-deprived (0.5% foetal bovine serum) for 24 h and then treated with 5 μM SAHA, 5 ng/ml TGFβ or the combination of the two. At 24 h, cells were lysed and analysed by IB with the indicated antibodies. **d** Total RNAs from HNC cell lines treated with 5 μM SAHA, 5 ng/ml TGFβ or the combination of the two, were isolated for RT-qPCR. PA-1, Jun, ITGB6 and vimentin expression was normalised to RpPO. Graphs show means ± SD of fold changes of expression of TGFβ- and TGFβ-SAHA-treated cells compared to vehicle-treated cells and SAHA-treated cells, respectively. *P < 0.05; **P < 0.01 (unpaired *t* test). Means are at least from three independent experiments

To understand the molecular mechanisms triggering the inhibitory effect of SAHA on HNC cell lines, we tested the ability of this drug in reverting the EMT phenotype, as already described in HNC HPV-negative cell lines.^16^ We confirmed these data also in HPV-positive cell lines (Fig. 1a, b), showing that SAHA was able to significantly increase the epithelial marker E-cadherin, both at mRNA and protein level, partially decreasing the protein expression of the mesenchymal marker vimentin. Moreover, as shown in figure S1,B, SAHA inhibited the activation of two main proliferative and migratory signalling pathways, such as PI3K and ERK1/2. SAHA was also able to decrease protein expression of the most abundant p63 isoform in these cell lines, ΔNp63α, in a post-transcriptional way (Fig. 1a, b), independently of the HPV status. As shown in Fig. 1a, b, UM-SCC-47 cell line does not express full-length ΔNp63α, due to the multiple integration of HPV16 at the *TP63* locus, leading to the expression of a truncated 25-kDa protein at the carboxyl terminus of p63.^44^ We then further investigated the role of SAHA in reverting EMT by stimulating HNC cell lines with TGFβ, which pathway is known to be upregulated during EGFR inhibition resistance.^12^ As shown in Fig. 1, SAHA was able to attenuate the effect of TGFβ by both reducing the activation of one of the main players of the TGFβ pathway, SMAD2 (Fig. 1c) and by blocking the transcription of some known TGFβ target genes (Fig. 1d) in both HPV-positive and HPV-negative cell lines. Moreover, as expected, TGFβ, alone or in combination with SAHA, had no effect on cell viability in both HPV-negative and -positive HNC cell lines with different sensitivity to SAHA (Figure S2B). We thus established the role of SAHA in reverting the transition to a more aggressive mesenchymal phenotype in these cell lines, which might be helpful in overcoming EGFR inhibition resistance.

### SAHA exerts its inhibitory activity through the reduction of ΔNp63α and EGFR expression

**Fig. 2:**
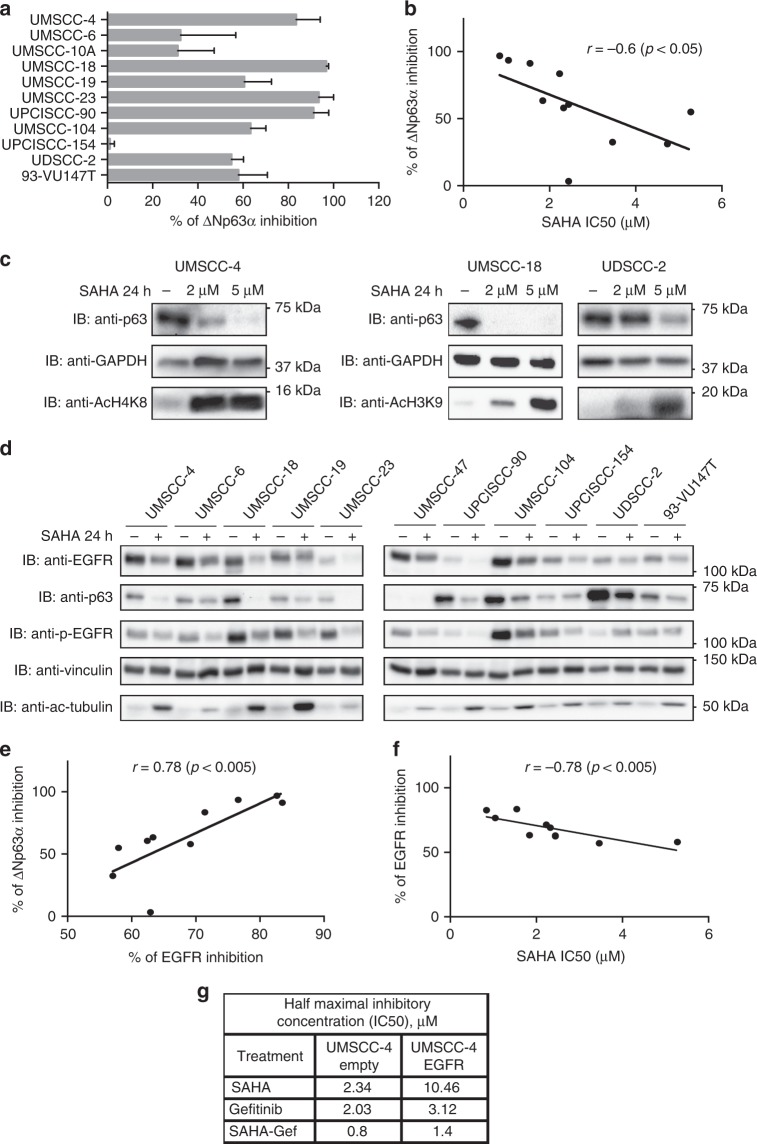
ΔNp63α downregulation is required for the inhibitory activity of SAHA, which treatment decreases epidermal growth factor receptor (EGFR) expression. **a** Optical density (OD) analysis of the expression of ΔNp63α from three independent western blot experiments of head and neck cancer (HNC) cell lines treated with 5 μM SAHA or vehicle for 24 h was performed. Results were normalised to loading control (GAPDH) and expressed as percentage of inhibition (means ± SD). **b** Percentage of ΔNp63α inhibition results obtained in **a** were correlated to SAHA IC50, Pearson correlation coefficient *r* = −0.6 (*P* < 0.05). **c** HNC cell lines were treated with 2 or 5 μM SAHA or vehicle for 24 h, lysed and analysed by immunoblotting (IB) with the indicated antibodies. **d** HNC cell lines were treated with 5 μM SAHA or vehicle for 24 h, lysed and analysed by IB with the indicated antibodies. **e**, **f** OD analysis of the expression of EGFR from three independent western blot experiments of HNC cell lines, shown in **d**, treated with 5 μM SAHA or vehicle for 24 h was correlated to OD results of percentage of p63 inhibition mediated by SAHA (**e**) or SAHA IC50 (**f**). Pearson correlation coefficient *r* = 0.78 (*P* < 0.005) and r = −0.78 (*P* < 0.005) respectively. **g** UM-SCC-4 cell line was transduced with EGFR-encoding viral particle; puromycin-selected cells were treated with SAHA, gefitinib and the combination of the two drugs, and IC50 was assessed using CellTiter-Glo^®^ Luminescent Cell Viability Assay

Since SAHA was able to reduce ΔNp63α expression in these cells, we further analysed whether this effect triggers the inhibitory effect of SAHA. We thus conducted a quantification analysis calculating the percentage of inhibition of p63 protein expression in all the cell lines (Fig. 2a) and compared it to the SAHA IC50 (Table 1), finding a great inverse correlation between the two values (Fig. 2b). These data were then confirmed by showing that in the cell line most sensitive to SAHA, UM-SCC-18, SAHA significantly decreased ΔNp63α expression at lower concentrations compared to the more resistant cell line UD-SCC-2, in which a higher concentration of SAHA was needed to considerably inhibit ΔNp63α expression (Fig. 2c). Indeed, in UM-SCC-4 cell line with a moderate sensitivity to SAHA the effect on ΔNp63α expression was halfway (Fig. 2c). Moreover, SAHA treatment was able to decrease the expression of the EGFR receptor both at mRNA and protein level (Fig. 2d and S1C), independently of the HPV status. In relation to this, we observed a great correlation between SAHA-mediated inhibition of both ΔNp63α and EGFR protein expression (Fig. 2e), together with an inverse correlation between SAHA IC50 and EGFR inhibition (Fig. 2f), suggesting that SAHA exerts its activity through the inhibition of the expression of both ΔNp63α and EGFR. In particular, these data were reinforced by the overexpression of EGFR in UM-SCC-4 cells, which caused a substantial increase in the IC50 of SAHA, gefitinib and the combination of the two drugs (Fig. 2g).

HDACi are able to decrease gene expression of both wild-type (wt) and mutant p53.^45^ Mutant p53 can control EGFR activity^46^ and increase its expression,^47^ while wt p53 loss increases EGFR expression.^48^ Thus, we checked the effect of SAHA on p53 expression in our cell lines. As shown in Figure S2A, SAHA is able to decrease p53 expression in HPV-positive cell lines with wt p53 (UM-SCC-47, UPCI:SCC-90, UM-SCC-104, UPCI:SCC-154 and UD-SCC-2), and also in 93-VU147T cells, which present a heterozygous mutation of p53 (Table S1). Only few HPV-negative cell lines express p53, among them UM-SCC-10A and UMSCC-23 cells, which mutated p53, also showed decreased expression of p53 upon SAHA treatment (Figure S2B and Table S1). We can conclude that in HNC cell lines SAHA decreases the expression of both wt and mutant p53; thus, the decreased expression of EGFR in our cell lines does not seem to be dependent on p53 expression and on its mutation status.

### p63 knockdown significantly decreases HNC cell line proliferation and migration

**Fig. 3:**
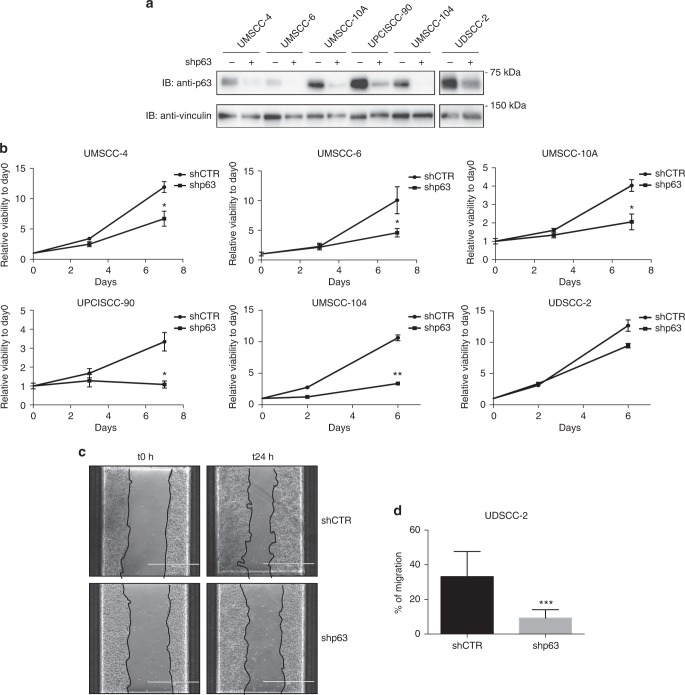
p63 knockdown decreased human papillomavirus (HPV)-positive and HPV-negative head and neck cancer (HNC) cell line proliferation and migration. **a** p63 short hairpin RNA (shRNA)-encoding lentiviral particles were used to transduce HNC cell lines; puromycin-selected cells were lysed and analysed by immunoblotting (IB) with the indicated antibodies. **b** Viability of p63 shRNA-transduced HNC cell lines was assessed using CellTiter-Glo^®^ Luminescent Cell Viability Assay and expressed as relative viability to the time point 0 (day 0) (means ± SD). *P < 0.05; **P < 0.01 (unpaired *t* test). **c** Phase contrast pictures of scratch wound-healing assay performed on UD-SCC-2 cells transduced with p63 or control shRNA at time 0 and 24 h after the scratch. **d** The graph represents the wound width measurements of p63 shRNA cells as compared with control shRNA cells and expressed as % of migration (means ± SD). ***P < 0.0005 (unpaired *t* test)

We then silenced p63 protein in both HPV-positive and HPV-negative cell lines using a lentiviral vector (Fig. 3a)^49^ and assessed cellular proliferation and migration. As shown in Fig. 3b, c respectively, lack of p63 expression consistently reduced cell proliferation and significantly decreased cell migration. With these data we confirmed a dominant role of p63 as a mediator of both proliferative and migratory pathway in HNC cell lines.

### Lack of p63 expression decreases EGFR expression and inhibits TGFβ-mediated EMT

**Fig. 4:**
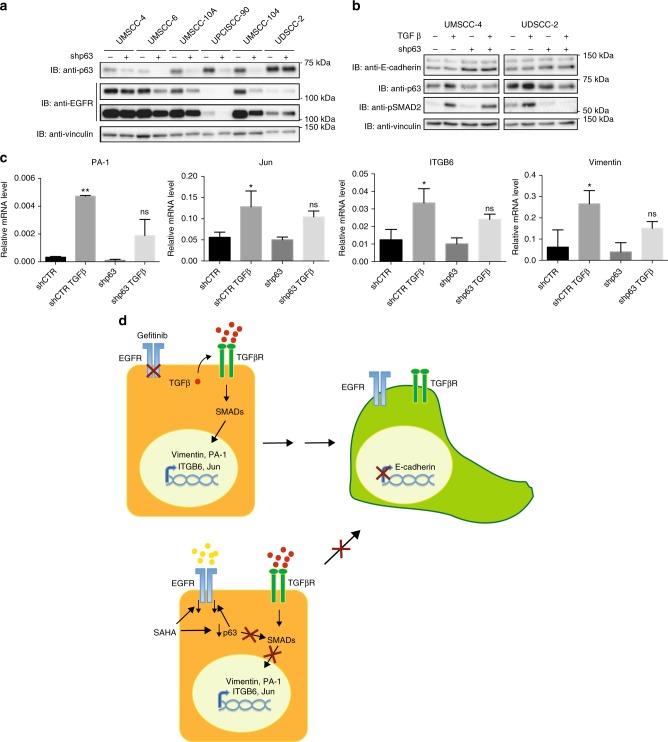
p63 knockdown decreases epidermal growth factor receptor (EGFR) expression and inhibits transforming growth facor β (TGFβ)-induced epithelial to mesenchymal transition (EMT) in human papillomavirus (HPV)-positive and HPV-negative head and neck cancer (HNC) cell lines. **a** p63- and control short hairpin RNA (shRNA)-transduced HNC cell lines were lysed and analysed by immunoblotting (IB) with the indicated antibodies. For the EGFR IB both the low and high exposures are shown. **b** p63- and control shRNA-transduced HNC cell lines were serum-deprived (0.5% foetal bovine serum) for 24 h and then treated with 5 ng/ml TGFβ. At 24 h, cells were lysed and analysed by IB with the indicated antibodies. **c** Total RNAs from UM-SCC-4 cell line transduced with p63 and control shRNA treated with 5 ng/ml, were isolated for RT-qPCR. PA-1, Jun, ITGB6 and vimentin expression was normalised to ribosomal phosphoprotein and expressed as means ± SD. *P < 0.05; **P < 0.01; ns not statistically significant compared to shCTR (multiple comparison one-way analysis of variance). **d** Schematic representation of the action of SAHA in reverting EMT process that causes gefitinib resistance. Gefitinb treatment may induce tumour-promoting TGFβ signals, which initiate EMT through activation of SMADs and upregulation of mesenchymal transcription factors. SAHA treatment decreases ΔNp63α, and subsequently downregulates EGFR expression and prevents the upregulation of TGFβ-dependent mesenchymal transcription factors by inhibiting SMAD activation

Recently, it has been shown that silencing endogenous ΔNp63α reduces EGFR expression in triple-negative basal-like breast cancer cells and in pancreatic cancer cells promoting cell growth and chemoresistance.^50,51^ We thus assessed EGFR expression in HPV-positive and HPV-negative HNC cell lines upon p63 knockdown. As shown in Fig. 4a, lack of p63 protein consistently decreased the protein level of EGFR. Moreover, decreased expression of p63 was sufficient to inhibit the ability of TGFβ to activate SMAD2 and promote target genes transcription (Fig. 4b, c, respectively). Taken together, these results showed the crucial role of p63 in regulating both EGFR expression and TGFβ pathway, confirming that p63 downregulation underlies the inhibitory activity of SAHA in these cell lines.

## Discussion

HNCs are a biologically heterogeneous group of cancers, originating from different subsites, mucosa of the oral cavity, nasopharynx, oropharynx, hypopharynx and larynx and with different aetiological factors, among them high-risk HPV infection and transformation. Thus, in our study we decided to collect different HNC cell lines, from different subsites, sex and HPV status, to have a better vision of the potential diversity of response to therapy. The cell lines that we obtained were not clearly clustered in subgroups by their expression of epithelial/mesenchymal markers or to the activation of different kinases important in the transduction of signalling pathways, confirming the high heterogeneity of these tumours. Although one of the signatures of these cancer is the overexpression of the EGFR, the inhibition of EGFR as main therapy has very modest efficacy due to intrinsic or acquired resistance.^8^ The HDACi SAHA was already shown to potentially overcome EGFR-TKI resistance in a small panel of HPV-negative HNC cell lines.^16^ In this study we decided to better investigate the efficacy of SAHA alone or in combination with gefitinib in a larger and heterogeneous panel of HNC cancer cell lines and to characterise the molecular mechanisms underlying its activity. We found that both SAHA and gefitinib were able to inhibit HNC cell proliferation in both HPV-positive and HPV-negative cell lines, with different sensitivity independently from either their HPV status or their epithelial/mesenchymal phenotype. Moreover, the combination of the two drugs had a synergistic effect on both HPV-positive and HPV-negative HNC cell lines. Intriguingly, we found that SAHA treatment was able to increase epithelial marker and partially reduce mesenchymal marker in HNC cell lines, independently of the HPV status. This confirms the role of this drug in reverting EMT also in HPV-positive cell HNC lines, a role that was reinforced by the ability of SAHA to inhibit the activation of the TGFβ pathway, one of the pathways responsible for EMT that is hyperactivated during the acquisition of gefitinib resistance.^12^

EGFR and ΔNp63α are two key markers in HNC since their expression is upregulated in 90% and 80% respectively of HNC primary tumours.^7,30^ They are both involved in the promotion of proliferation and migration of HNC cell lines, cooperating with the TGFβ pathway or independently, activating pathways such as PI3K and MAPK.^12^ Upon SAHA treatment, we found a remarkable decrease in the expression of both ΔNp63α and EGFR in both HPV-positive and HPV-negative HNC cell lines, together with the inhibition of PI3K and ERK1/2 pathways, and, as mentioned above, decreased activation of the TGFβ pathway. Thus, our results suggest that the inhibition of both EGFR and ΔNp63α expression is responsible for the inhibitory activity in cell proliferation and migration mediated by SAHA. This evidence is reinforced by the remarkable inverse correlation between SAHA sensitivity and SAHA-mediated inhibition of EGFR and ΔNp63α. This behaviour also mirrors the positive correlation between ΔNp63α and EGFR inhibition by SAHA and the less efficacy of SAHA in cells overexpressing EGFR. In particular, ΔNp63α is known to regulate EMT in primary human keratinocytes and in HNC cell lines in a TGFβ-dependent manner^33,34^ and its downregulation reduces EGFR expression in triple-negative basal-like breast cancer cells and in pancreatic cancer cells promoting cell growth and chemoresistance.^50,51^ Thus, we showed for the first time that p63 silencing reduced EGFR expression in both HPV-positive and HPV-negative cell lines, showing also that SAHA-mediated inhibition of EGFR greatly correlates with ΔNp63α inhibition. Taken together, these data suggest that SAHA-mediated EGFR downregulation is p63-dependent. Moreover, since lack of p63 consistently reduced proliferation and migration of both HPV-positive and HPV-negative cell lines, interfering with the activation of the TGFβ pathway, our results imply a key role  for p63 in the SAHA-dependent regulation of proliferation and migration of these cell lines. In conclusion, we found that not only the HDACi SAHA synergises with gefitinib to decrease HNC cell lines viability, but it is also able to reduce EMT and inhibit TGFβ pathway activation, which are responsible for the induction of gefitinib resistance. A key regulator of this process is the transcription factor ΔNp63α, whose downregulation by SAHA treatment appears to be the major inhibitory activity of this drug, decreasing cell growth, cell migration and TGFβ pathway activation. Thus, this study uncovers a novel molecular mechanism underlying the efficacy of SAHA in the treatment of both HPV-positive and HPV-negative HNC tumours in combination with gefitinib. Gefitinib efficacy in the treatment of these tumours is limited due to the acquired resistance induced by EMT, which we demonstrated can be overcome by the concomitant use of SAHA. These results show that the combination of SAHA with specific inhibitors of EGFR, such as gefitinib, improves the antitumour efficacy of these drugs in both HPV-positive and HPV-negative HNC and should be further explored clinically.

## Supplementary information

Supplementary Figure S1

Supplementary Figure S2

Supplementary Figure legends

Supplementary Table S1

## Data Availability

All relevant data are within the paper and its Supporting Information files.
