# Supplementary material for: Synergistic antitumour activity of HDAC inhibitor SAHA and EGFR inhibitor gefitinib in head and neck cancer: a key role for ΔNp63α
Source: Br J Cancer. 2019 Feb 15;120(6):658–67. doi: 10.1038/s41416-019-0394-9 (PMC6461861; doi:10.1038/s41416-019-0394-9)
Supplement: Supplementary file 3 — Supplementary Figure legends [file 41416_2019_394_MOESM3_ESM.docx]

Figure S1. (A) HNC cell lines were lysed and analysed by IB with the indicated antibodies.

(B) HNC cell lines were treated with 5 μM SAHA or vehicle for 24h, lysed and analysed by

IB with the indicated antibodies. (C) Total RNAs from HNC cell lines treated with 5 μM

SAHA or vehicle for 24h were isolated for RT-qPCR. EGFR expression was normalized to

RpPO and expressed as means ±SD of at least three independent experiments. *, P <

0.05 (Unpaired t test).

Figure S2. (A) HNC cell lines were treated with 5 μM SAHA or vehicle for 24h, lysed and

analysed by IB with the indicated antibodies. (B) The represented HNC cell lines were

treated with 5 μM SAHA, 5 ng/ml TGFβ or the combination of the two for 24h. Cell viability

was then assessed using CellTiter-Glo® Luminescent Cell Viability Assay and expressed

as percentage of cell viability relative to untreated cells (means ±SD). *, P < 0.05; **, P <

0.01; ****, P < 0.0001; ns, not statistically significant compared to control (multiple

comparison one-way ANOVA).
