# Supplementary material for: Synergistic antitumour activity of HDAC inhibitor SAHA and EGFR inhibitor gefitinib in head and neck cancer: a key role for ΔNp63α
Source: Br J Cancer. 2019 Feb 15;120(6):658–67. doi: 10.1038/s41416-019-0394-9 (PMC6461861; doi:10.1038/s41416-019-0394-9)
Supplement: Supplementary file 4 — Supplementary Table S1 [file 41416_2019_394_MOESM4_ESM.docx]

| Panel of HNC cell lines used in the study |
| --- |

CELL LINES Subsite of origin Sex HPV status TP53 mutation

|  |
| --- |

UMSCC-4 Oropharynx F - 213 stop

UMSCC-6 Oropharynx M - unknown

UMSCC-10A Larynx M - G245C

UMSCC-18 Oropharynx M - unknown

UMSCC-19 Oropharynx M - 148 frameshift

UMSCC-23 Larynx F - C176F

UMSCC-47 Oropharynx M + wt

UPCISCC-90 Oropharynx M + wt

UMSCC-104 Oral cavity M + wt

UPCISCC-154 Oral cavity M + wt

UDSCC-2 Hypopharynx M + wt

93-VU147T Oral cavity M + L257R/wt
